# Members of the League of Mercy at Marlborough House

**Published:** 1903-05-30

**Authors:** 


### HOSPITAL ADMINISTRATION.

### CONSTRUCTION AND ECONOMICS.

### MEMBERS OF THE LEAGUE OF MERCY AT MARLBOROUGH HOUSE.

On the afternoon of Friday, the 22nd inst., their Royal Highnesses the Prince and Princess of Wales received the members of the League of Mercy at Marlborough House. At half-past three the presidents and lady presidents of the league, to the number of about 100, assembled in the newly decorated saloon, where Mr. J. Harrison, M.V.O., one of the hon. secretaries, presented to their Royal Highnesses, as Grand Presidents of the League, the report for the year. It was also announced that Lady Pirbright had sent a donation of £500 to the League in memory of Lord Pirbright, who was president of the Guildford district. The registrar of the League, Colonel Knollys, then read a list of those to whom the Order of Mercy had been awarded by the King, and the Prince of Wales cordially thanked the presidents and other workers of the League, and expressed the pleasure which he felt at meeting personally those whose efforts had made its organisation and work so successful.

After this the Royal party and the ladies and gentlemen who were at the meeting adjourned to the garden, where about 1,200 of the vice-presidents and members of the League were assembled. The Princess, who wore a becoming gown of pale hyacinth blue, with a toque of flowers of the same tint, was accompanied by Princes Edward and Albert, and by Princess Mary. The little Princes were dressed in white sailor-suits and shady straw-hats, and Princess Mary, who has inherited the Princess's delicate complexion and curling golden hair, wore a simple white dress and hat. As the Royal party came out the band of the Royal Marines, Chatham, played the National Anthem, and their Royal Highnesses acknowledged the salutations of their guests before taking their stand by a table on which lay the Orders to be presented. The ladies and gentlemen who were to be thus honoured then passed before their Royal Highnesses. The Prince, as he handed the cross and ribbon of the Order of Mercy in its case, shook hands with each recipient. The Princess also shook hands with those who were personally known to her, and courteously acknowledged the salutations of all. The Royal children, standing by their mother's side, looked with interest on the scene and seemed gratified that some of the ladies, after having curtsied to the Princess, made a like obeisance to them.

The ladies and gentlemen who were invested with the Order of Mercy were :—

Her Royal Highness the Duchess of Albany (Lady President, Deptford).
The Countess Grosvenor (Lady President, St. George's, Hanover Square).
The Dowager Lady Lamington (Lady President, St. Pancras, North).
The Lady Evelyn Ewart (Lady President, Maldon).
The Hon. Mrs. Freeman Thomas (Lady President, Eastbourne).
Lady Burdett (Lady President, Paddington, South).
Mrs. Seymour Corkran (Lady President, Westminster).
Mrs. Lockwood (Lady President, Epping).
Mrs. Boulnois (Lady President, Marylebone, East)
His Grace the Duke of Argyll, K.T., G.C.V.O. (President, Kensington, South).
The Lord Farquhar, G.C.V.O. (President, Marylebone, West).
Lady Howard Vincent (Lady Vice-President, St. George's, Hanover Square).
The Hon. Mrs. Leveson-Gower (Lady Vice-President, Reigate).
Lady Edridge (Lady Vice-President, Croydon).
Lady Hooker (Lady Vice-President, Chertsey).
Mrs. Brand (Lady Vice-President, Sevenoaks).
Mrs. Bright (Lady Vice-President, Maldon).
Mrs. Brown (Lady Vice-President, Walthamstow)
Miss Campbell (Lady Vice-President, Lambeth, Norwood).
Miss Laura Devas (Lady Vice-President, Sevenoaks).
Mrs. Fleming (Lady Vice-President, Sevenoaks).
Mrs. John Gabriel (Lady Vice-President, Lambeth, Norwood).
Mrs. Gandy (Lady Vice-President, Lambeth, Norwood).
Mrs. Newman Gilbey (Lady Vice-President, Epping).
Mrs. F. E. Gladstone (Lady Vice-President, Fulham).
Mrs. Harold Hardy (Lady Vice-President, Marylebone, West).
Mrs. Horan (Lady Vice-President, Tonbridge)
Mrs. Johnston (Lady Vice-President, Sevenoaks).
Mrs. Keay (Lady Vice-President, Eastbourne).
Mrs. Wingfield King (Lady Vice-President, Kingston and Richmond).
Miss Maclean (Lady Vice-President, Kingston and Richmond).
Mrs. Metcalfe (Lady Vice-President, Paddington, South).
Miss Ethel H. Montgomery (Lady Vice-President, Chelsea).
Mrs. Smart (Lady Vice-President, Southwark, Bermondsey).
Miss Trouncer (Lady Vice-President, Kingston and Richmond).
Mrs. Woolland (Lady Vice-President, Fulham).
Gen. L. V. Lloyd (Vice-President, Westminster).
Col. Wilford Loyd (Vice-President, St. George's, Hanover Square).
Lieut.-Col. W. Narborough, V.D. (Vice-President, Woolwich).
Col. L. Worthington Wilmer (Vice-President, Fulham).
Frederick T. Aston, Esq. (Vice-President, Kingston and Richmond).
Charles Davis, Esq. (Vice-President, Marylebone, West).
Norman Hay Forbes, Esq., F.R.C.S.Ed. (Vice-President, Tonbridge).
Clement Godson, Esq., M.D. (Vice-President, St. George's, Hanover Square).
Herbert Hordern, Esq. (Vice-President, Faversham).
T. F. McDonnell, Esq., F.R.C.P.I. (Vice-President, Eastbourne).
Frank Manby, Esq. (Vice President, Fulham).
W. R. Smith, Esq. (Vice-President, Deptford).
E. Spicer, Esq. (Vice-President, Kensington, South).
Arthur Williams, Esq. (Vice-President, Fulham).
Holland Hodgson Wright, Esq., M.R.C.S. (Vice-President, St Pancras, North).
Mrs. H. C. Marshall (Member, Marylebone, East).
Miss E. R. Scott (Member, Marylebone, East).
Miss E. J Seligman (Member, Kensington, South).
Mrs. Temperley (Member, St. George's, Hanover Square).
The Hon. Sir Raymond Robert Tyrrwhitt Wilson, Bart. (Member, St. George's, Hanover Square).
J. M. Freshwater, Esq. (Member, City of London).
F. Voelklein, Esq. (Member, St. George's, Hanover Square).

All of these, however, were not able to be present to receive it in person.

Among the presidents, vice-presidents, and members of the League who were present, besides those who were that day invested with the Order, were Countess Cadogan, Georgina Countess of Dudley, Lady Llangattock, Lord and Lady Rothschild, Sir Savile and Lady Crossley, Sir William and Lady Collins, Sir Joseph and Lady Dimsdale, Sir John and Lady Aird, Lady Faudel Phillips, Mr. and Mrs. Leopold de Rothschild, Sir Henry and Lady Burdett, Mrs. Halford Burdett, Mrs. R. L. Harrison, and others. Many people looked with interest at a sturdy man in a blue railway uniform who was going about with the aid of a stick. This was Mr. Michael Cahill, a member of the Westminster division of the League, and a devoted worker for it, in spite of the fact that his activity is hampered by both his legs being artificial. He can boast of only one sound knee, but, crippled as he is, he can work in the cause of charity, and he had been honoured with an invitation to the palace of the Grand President.

After the investiture, the Prince and Princess walked through the grounds and conversed with some of their personal friends, while others of the guests had the honour of being presented to them. Refreshments had been provided in a long tent draped with delicate white and green, while the tables were beautified with high bouquets of great pink roses and Madonna lilies. Another tent, with small tables set round with gilt chairs, accommodated the Royal hosts and their more favoured guests, and when the Prince and Princess went in to tea Lady Lamington, who was in attendance on the Princess, brought little Prince Henry from the house to join his parents, while the youngest member of the Royal family surveyed the gay scene from the window of his nursery.

After tea the Prince and Princess again went in and out among their guests, conversing with many of them, until the band played "God Save the King," and the guests went away gratified by the Royal graciousness and encouraged by it to continue their labours in the cause of the sick and suffering.

